# The learning progression of diagnostic sialendoscopy^☆^^☆☆^

**DOI:** 10.1016/j.bjorl.2015.10.007

**Published:** 2015-11-06

**Authors:** José Higino Steck, Elaine Stabenow, Erivelto Martinho Volpi, Evandro Cezar Guerreiro Vasconcelos

**Affiliations:** a Cirurgia de Cabeça e Pescoço, Clínica ONCCAPE, Campinas, SP, Brazil; b Serviço de Cirurgia de Cabeça e Pescoço do Hospital das Clínicas da Faculdade de Medicina da Universidade de São Paulo (HCFM-USP), São Paulo, SP, Brazil

**Keywords:** Salivary gland diseases, Endoscopy, Diagnosis, Doenças da glândula salivar, Endoscopia, Diagnóstico

## Abstract

**Introduction:**

Sialendoscopy is becoming the gold standard procedure for diagnosis and treatment of Salivary Gland Inflammatory Diseases.

**Objective:**

To evaluate the learning progression of a single surgeon to implement and perform diagnostic sialendoscopy: to estimate how many procedures were necessary to achieve better results; if it was higher rate of complications in the beginning.

**Methods:**

Retrospective analysis involving 113 consecutive sialendoscopies performed from 2010 to 2013. According to a descriptive analysis of the factors related to surgeon's experience, the casuistic was divided into two groups: group (A) comprising the first 50 exams, and group (B) the last 63. Groups were then compared concerning demographic and peri-operative aspects.

**Results:**

In Group A, failure to catheterize papilla were 22% *versus* 3% in B (*p* = 0.001). Failure to complete examination was 30% in group A *versus* 6% in B (*p* = 0.001), and necessity to repeat exams was 22% in group A *versus* 10% in B (*p* = 0.058). The complication rates were 18% in group A, and 10% in B (*p* = 0.149). Operative time was slightly shorter in group B (56 *versus* 41 min, *p* = 0.045).

**Conclusion:**

We found better outcomes after the first 50 diagnostic sialendoscopies. Complication rates were statistically the same between early and late groups of experience with sialendoscopy.

## Resumo

**Introdução:**

A sialoendoscopia vem se tornando o procedimento de referência para o diagnóstico e o tratamento das doenças inflamatórias da glândula salivar.

**Objetivo:**

Avaliar a progressão de aprendizado de um mesmo cirurgião para implementação e realização da sialoendoscopia diagnóstica: verificar quantos procedimentos foram necessários para a obtenção de resultados melhores e se houve ocorrência de maior taxa de complicações no início do aprendizado.

**Método:**

Análise retrospectiva envolvendo 113 sialoendoscopias consecutivas realizadas de 2010 a 2013. De acordo com uma análise descritiva dos fatores relacionados à experiência do cirurgião, a casuística foi dividida em dois grupos: grupo (A), compreendendo os primeiros 50 exames; e grupo (B), os últimos 63. Em seguida, os grupos foram comparados, levando em consideração os aspectos demográficos e perioperatórios.

**Resultados:**

No grupo A, a não realização do cateterismo papilar foi de 22% *vs.* 3% em B (p = 0,001). A não realização de um exame completo foi de 30% no grupo A *vs.* 6% em B (p = 0,001), e a necessidade de repetir o exame foi de 22% no grupo A *vs.* 10% em B (p = 0,058). Os percentuais de complicações foram 18% no grupo A e 10% em B (p = 0,149). O tempo operatório foi ligeiramente menor no grupo B (56 *vs.* 41 minutos, p = 0,045).

**Conclusão:**

Verificamos melhores desfechos após as 50 primeiras sialoendoscopias diagnósticas. Os percentuais de complicações foram estatisticamente semelhantes entre os grupos inicial e avançado de experiência com a sialoendoscopia.

## Introduction

In the last 15 years the gold standard in the treatment of Salivary Gland Inflammatory Diseases (SGID) moved from open surgical resection to endoscopic treatment, with the obvious gain of avoiding complications from parotid and submandibular surgery, like scars and nerve injury, and preserving salivary function.^1^, ^2^ Sialendoscopy was first described for the treatment of sialolithiasis.^3^, ^4^ Rapidly the technique gained space in the diagnosis and treatment of other SGID, like Radioiodine sialadenitis, Sjogrens disease and Juvenile recurrent parotiditis.^5^ However, the use of very delicate instruments in very small and delicate structures as the salivary papillae and ducts proved to be very challenging.^6^ In 2006, Marchal et al.^7^ were working yet to improve the sialendoscopy technique to enter the small branches of the salivary ducts.

As the benefits of the sialendoscopy in regard to the open surgeries became evident,^1^, ^2^ some training centers were established, as the European Sialendoscopy Training Center, created in 2002 by Francis Marchal.^1^ Despite a growing experience about this procedure, some important doubts remain, such as whether the complication rate is higher at the beginning of the learning curve and how many cases are necessary for a surgeon to perform a safe sialendoscopy.^8^, ^9^ Thus, the objective of this paper is to review and evaluate the learning progression of a single surgeon to implement and perform diagnostic sialendoscopy in both parotid and submandibular glands, in order to estimate how many procedures were necessary to achieve good results and if the rate of complications was higher in the beginning.

## Methods

This is a retrospective, transversal, consecutive-cases study. It was approved by the Institutional Review Board (CO003), and all patients gave their informed consent prior to their inclusion in the study.

All patients submitted to sialendoscopy performed by the same surgeon from September 2010 to January 2013 were included. Parotid or submandibular exams were all included. There was no exclusion criterion. Accordingly, the casuistic comprises 113 sialendoscopies concerning 65 patients (62% females) with a mean age of 46 years (ranging from 4 to 83). Concerning the examined glands, 45% were parotid and 55% submandibular glands.

The surgeon had an initial hands-on training on fresh pig head specimens at the ESTC – European Sialendoscopy Training Centre, Geneva, Switzerland.

All patients initially undergone a diagnostic sialendoscopy.

Patients were placed in supine or semi-seated position, under local or general anesthesia. We utilized Marchal diagnostic and interventional miniature endoscopes (Karl Storz™) with an external diameter 1.1 or 1.3 mm, a micro camera and video system.

**Figure 1:**
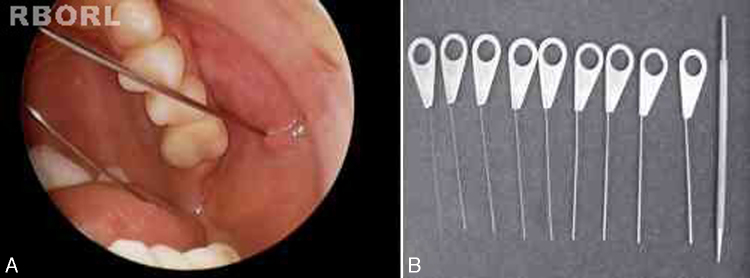
Papilla catheterization of a left parotid gland (A) and probes utilized to perform papilla dilation (B).

All sialendoscopies started with location, catheterization and dilation of the salivary papilla with probes (Fig. 1). When papilla catheterization was achieved, the sialendoscope was introduced through the papila, while the salivary duct was irrigated with saline solution or distilled water to allow video screen visualization. Endoscopic findings were carefully studied and measured for a detailed description: ductal stenosis, salivary stones, chronic sialadenitis signs (pale mucosa, stenosis and plugs of mucus), acute sialadenitis signs (friable and reddish mucosa with pus) and ductal plugs of mucus due to stasis of saliva flow.

**Figure 2:**
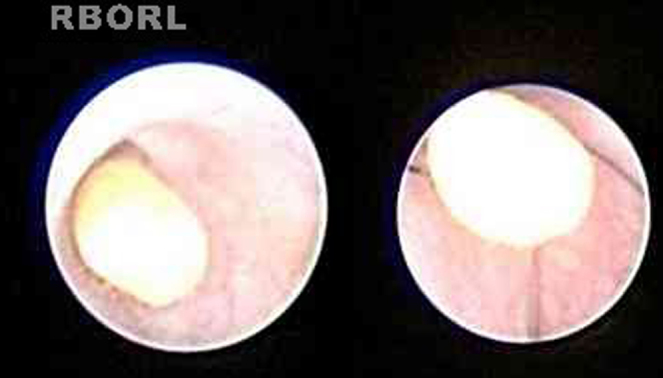
Sialendoscopic view of a stone and its removal with mini basket catheter.

In some cases, treatment was performed in the same procedure, according to the endoscopic finding, like stone removal by basket (Fig. 2), fragmentation by laser lithotripsy or both. Stenosis was treated with endoscopic dilation. Intraductal instillation of 100 mg of hydrocortisone was systematically done at the end of the all procedures.

Postoperative care followed a Fast Track Rehabilitation routine including opioids free analgesia, immediate postoperative feeding and ambulation. All sialendoscopies were executed in an outpatient fashion.

In order to estimate how many procedures were necessary to achieve good results, an initial analysis of surgeon-related outcomes was performed based on medical chart review. We searched caracteristics that allowed dividing the casuistic in early and late groups of sialendoscopy experience.

Demographic and the following perioperative aspects were analyzed: (1) the duration time of sialendoscopies in minutes; (2) failure in papilla catheterization; (3) failure to complete the endoscopic examination; (4) necessity to repeat sialendoscopy in the same patient; (5) failure to achieve therapeutic goal; (6) perioperative complication rates.

Papilla was considered successfully catheterized when it was possible to find it and introduce at least one dilation probe inside.

Examination was considered successful when it achieved progression of the endoscope (external diameter 1.1 or 1.3 mm) to at least one ramification of the main duct or until disease was found.

Treatment was considered successful when the therapeutic goal was achieved, as stone removal or stenosis dilation.

### How groups were defined

Visual analysis of graphically displayed data suggested that surgeon-related outcomes improved around the 50th sialendoscopy.

Papilla catheterization was strongly related to surgeon's experience and was an important step because the diagnostic procedure is possible only if it is achieved.

**Figure 3:**
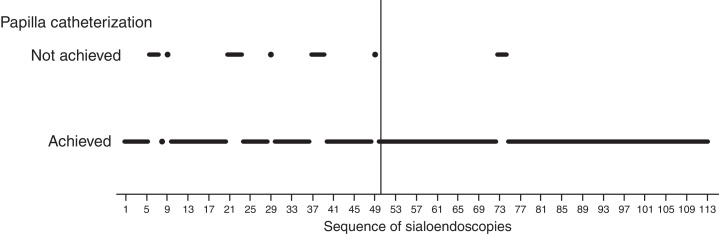
Sequence chart of 113 consecutive sialendoscopies. Black points at lower horizontal position represent procedures in which papilla catheterization was achieved, while those at upper position represent failure to achieve it. Vertical dashed line marks the 50th procedure.

The 50th sialendoscopy (Fig. 3) was the last one of the sequence when failure to achieve papilla catheterization was frequent (22%).

After the 50th sialendoscopy, only in two procedures (3%) papilla catheterization was not achieved (Fig. 3). They were performed in bilateral parotid glands of a 28 year-old woman who received radioiodine therapy for papillary thyroid carcinoma. There was a huge papilla stenosis suggesting anatomic difficulties more than an inexperienced surgeon.

Based on these findings, two groups were defined: early group (A) of the sialendoscopy surgeon's experience, comprising the first 50 exams, and late group (B) with the last 63 sialendoscopies.

### Statistics

Statistical analysis was undertaken using the SPSS software, version 19.0 (SPSS, Chicago, IL).

Before describing or comparing the study groups, Kolmogorov–Smirnov Test of Normality was performed for continuous variables. Fisher exact test was used to compare frequencies. Student's *t*-test was used to compare means. Wilcoxon Signed Ranks Test was used to compare paired non-parametric distributions. *p*-Value less than 0.05 was considered as statistically significant.

### Ancillary analysis

**Figure 4:**
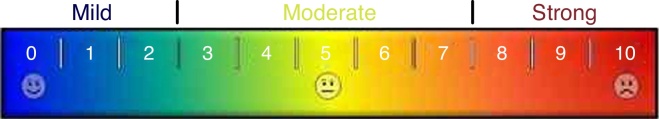
Visual color scale used to score the sialadenitis symptoms.

It was also made a comparison between paired pre- and postoperative visual color scale scores of sialadenitis symptoms that were filled out by 21 latest patients. Those cases were submitted either to diagnostic or diagnostic and therapeutic sialendoscopies. Visual color scale scores zero for absence of symptoms and 10 for maximal symptomatic disease (Fig. 4). Visual color scale questionnaires were applied in an outpatient fashion, before sialendoscopy and around the 30th postoperative day.

## Results

### Comparing early and late groups of the sialendoscopy experience

The patients mean age was similar between groups: group A 49 ± 3, ranging from 17 to 83 years old; group B 43 ± 4 years old, ranging from 4 to 75 (*p* = 0.21).

Females were more frequent in both groups: 58% in group A and 65% in group B (*p* = 0.38).

In group A submandibular gland sialendoscopies were more frequent (74%) than in group B (40%; *p* = 0.0001*).

Sialadenitis symptoms without stone were the most frequent finding in both groups (Table 1). There was more salivary stone in group A. Group B had more patients with history of radioiodine treatment and with recurrent juvenile parotitis.

**Table 1:** Sialendoscopy indications of early and late groups of sialendoscopy experience.

	Early group – A (*n* = 50)	Late group – B (*n* = 63)	Total
Salivary stone^a^	13 (26%)	10 (16%)	23 (20%)
Other obstructive sialadenitis^a^	26 (52%)	22 (35%)	48 (43%)
Radioiodine induced sialadenitis^a^	2 (4%)	13 (21%)	15 (13%)
Sjogren syndrome	5 (10%)	6 (9%)	11 (10%)
Recurrent juvenile parotitis^a^	1 (2%)	6 (9%)	7 (6%)
Acute sialadenitis	2 (4%)	4 (7%)	6 (5%)
Obstruction after open-surgery	1 (2%)	2 (3%)	3 (3%)

US, ultrasonography.

a Fisher's exact test, *p* ≤ 0,05.

General anesthesia was performed in 80% of group A and 89% of group B (*p* = 0.147).

**Figure 5:**
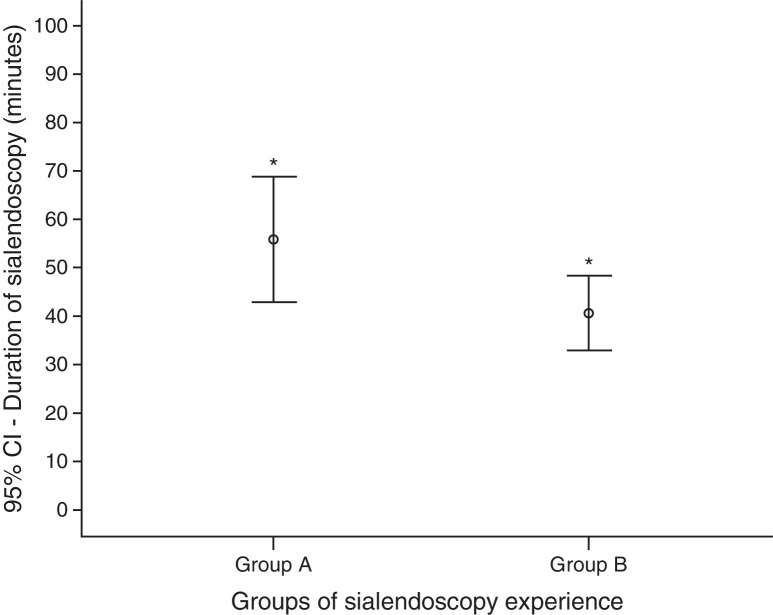
Comparison between means of sialendoscopy duration (min) according to early and late groups of surgeon experience (*Student *t*-test, *p* = 0.045).

The duration time of sialendoscopies in group A was higher than group B: mean of 56 min (ranging from 20 to 160) against 41 min (10–120) (*p* = 0.045) (Fig. 5). To analyze the sialendoscopy duration for each examined gland, we excluded cases in which papilla catheterization was not achieved.

Table 2 displays the comparison of perioperative outcomes between groups A and B. There was higher frequency of failure of diagnostic sialendoscopy in group A mainly due to failure of papilla catheterization in this group. When the papilla was successfully catheterized, failure to complete endoscopic examination was higher in group A too. Therefore, necessity to repeat this procedure in the same patient in group A was more frequent.

**Table 2:** Comparison of perioperative outcomes between groups of early and late sialendoscopy experience.

	Early group – A (*n* = 50)	Late group – B (*n* = 63)	Total	*p*
Failure of diagnostic sialendoscopy	15 (30%)	4 (6%)	19 (17%)	0.001^a^
Failure of papilla catheterization	11 (22%)	2 (3%)		
Failure to complete examination	4 (8%)	2 (3%)		
Necessity to repeat sialendoscopy	11 (22%)	6 (10%)	17 (15%)	0.058
Perioperative complication	9 (18%)	6 (10%)	15 (13%)	0.149

a Fisher's exact test statistically significant.

Group A sialendoscopies with failure to complete examination included the 11 procedures where papilla catheterization was not achieved and 4 others; false passage of the endoscope due to duct perforation; sialendoscopy under local anesthesia interrupted by pain; two others due to anatomic difficulties.

Group B sialendoscopies with failure to complete examination included the 2 procedures where papilla catheterization was not achieved and two others; previous resection of the accessory parotid gland; extensive duct fibrosis with perforation and false passage of the endoscope.

As in group A submandibular gland sialendoscopies were more frequent, we performed a multivariate analysis and found that, independent of the gland type, sialendoscopies of group A were related to more failures to complete examination (multivariate test, *p* = 0.02).

Endoscopic findings of the sialendoscopies are displayed in Table 3.

**Table 3:** Endoscopic findings in early and late groups of sialendoscopy experience (excluded cases with failure to complete sialendoscopy).

	Early group – A (*n* = 35)	Late group – B (*n* = 59)	Total
Lithiasic disease	12 (34%)	11 (19%)	23 (25%)
Ductal stenosis	11 (31%)	20 (34%)	31 (33%)
Chronic sialadenitis signs	3 (9%)	12 (20%)	15 (16%)
Acute sialadenitis signs	2 (6%)	4 (7%)	6 (6%)
Only ductal plugs of mucus	2 (6%)	9 (15%)	11 (12%)
Normal aspects	5 (14%)	3 (5%)	8 (8%)

Treatment was intended in 70% (36/50) of the procedures in group A and in 91% (57/63) in group B (*p* = 0.006). Failure to achieve treatment in group A happened in 25% (9/36), and in group B in 5% (3/57) (*p* = 0.008).

There was no statistically significant difference in perioperative complications between groups (Table 2). The complications were the following:

False passage of the endoscope due to duct perforation: 3 in group A and 4 in group B;
Papilla avulsion: 2 in group A and 1 in group B;
Severe swelling and pain symptoms associated with glandular infection in 1 case of each group;
Salivary fistula in 1 case of group A;
Salivary duct restenosis in 2 cases of group A.

### Ancillary analysis

Preoperative median score of the visual color scale of sialadenitis symptoms (9 ranging from 5 to 10) was higher than the postoperative one (2 ranging from 0 to 9) (Wilcoxon Test, *p* = 0.001). Even patients submitted to diagnostic sialendoscopy without a treatment procedure presented a relief of sialadenitis symptoms due to papilla dilation and steroids instilation.

## Discussion

Sialendoscopy is a relatively new procedure for the diagnosis and treatment of salivary duct diseases. It was introduced into clinical practice in the late 1990s, and caused an expressive change in the diagnostic and therapeutic management of sialolithiasis.^3^ One of the major changes was the reduction of the number of salivary glands removed because of salivary gland stones.^4^

Until recently, the main use of sialendoscopy was to confirm the diagnosis of obstructions and to remove sialoliths, but its use was extended to other inflammatory pathologies like radioiodine sialadenitis, juvenile recurrent parotitis, Sjogren's disease and duct stenosis.^10^, ^11^, ^12^, ^13^

Sialendoscopy is very helpful as a diagnostic method in the investigation of inflammatory diseases of unknown origin within salivary glands.^14^ Despite its conceptual simplicity, sialendoscopy is a challenging procedure, particularly the therapeutic ones, requiring skilled and experienced hands given the potential risk of perforation of the salivary duct.^2^

Obstacles to implement this procedure include the initial cost of the equipment and the associated learning curve of using a meticulous new technique.^6^

In this study, most of endoscopies (85%) were performed under general anesthesia in an outpatient fashion, but the procedure can be performed under local anesthesia too. The type of anesthesia can be a matter of choice for the patient or the surgical team, but it seems that general anesthesia can be better suitable for the beginning of the learning curve and for complex cases too.

Sialolithiasis is considered in the literature the main cause of obstructive salivary diseases, responsible for 66% of cases.^13^ In our casuistic, however, it was a less common cause (23%). One of the reasons for that is the presence of other common causes of obstructive sialadenitis less described in literature series but common in our clinical practice, like radioiodine induced sialadenitis (13%), because we are a reference center for thyroid cancer treatment. Stenosis without lithiasis is a common cause of obstruction in our experience (31%), sometimes with a known cause (like radioiodine or Sjogren disease), and sometimes with no apparent cause (idiopathic). The literature considers stenosis as the second cause of obstruction.^13^ With the development of the sialendoscopy technique, probably indications other than lithiasis will be more and more frequent. Accordingly, in our experience RIT induced sialadenitis was more frequent in group B. Despite the variability of causes for indication of sialendoscopy, the technique for the diagnostic exam is the same, so our analysis of the learning progression can be extrapolated.

All the procedures were performed by the same surgeon with the same equipment after initial recognized hands on training. Postoperative outcomes that were better after 50 sialendoscopies are linked to direct surgeon ability, especially salivary papilla catheterization.

Analyzing interquartile charts of sialendoscopies sequence, it was noticed that the first two quartiles concentrated more cases of failures in papilla catheterization, cases of failure in completing the examination and achieving treatment goal when compared to late quartiles. This improvement was statistically confirmed by comparisons between early and late groups, both in univariate and multivariate analysis. This data suggests that 50 procedures could be considered as an acceptable limit in the learning curve for sialendoscopy. After the first 50 exams papilla catheterization was not achieved only in bilateral parotid glands of the same patient, suggesting a specific anatomical variation, rather than an unexperienced surgeon.

In the literature, Danquart et al.^15^ described that the success rates rose from the first 25 procedures to the final 25 procedures, although not significantly. So it seems that the learning curve effect is really obtained after more than 25 procedures, as we found.

The groups’ demographic and clinical features were homogeneous, but more frequent submandibular endoscopies were found in the initial group. We asked if the worst outcomes in the beginning could be due to this finding and not due to learning curve. To answer this question a multivariate analysis was performed. Results showed better outcomes with the learning progression independently of the frequency of the examined gland. The submandibular endoscopy was only associated with more complications than parotid gland, while the learning curve did not affect the complication rates.

The operative time was shorter in group B, even with more complete exams and more therapeutic procedures, which demand more time. In group A the sialendoscopy mean duration was 56 min, and in group B 41 min. The literature describes that the mean duration of diagnostic and operative sialendoscopy is, respectively, 26 ± 14 and 73 ± 43 min.^16^

Papilla catheterization was achieved in 89% of the procedures, similar to literature.^15^ More experienced groups describe success rates from 96% to 98%^17^, ^18^ to achieve therapeutic goal, the same as group B (95%), while group A had only 75%.

Despite the surgeon learning curve, the overall rate of complications was low, comparable with literature.^19^

The most frequent effect of sialendoscopy is the transitory glandular swelling due to irrigation. The frequency in our study was 61%, and literature describes as high as 80–100%.^13^

Major complications as duct perforation with false passage were rarely described in the literature. Our incidence was documented as 8%, all with spontaneous resolution. Danquart^15^ reported 6%. Most of our complications were in submandibular exams. We also had two cases of infection and we decided to systematically prescribe antibiotics after that. In the literature the frequency described was 1.6%.^1^ Other complications described in literature^1^ like bleeding and nerve injury were not observed in our casuistic. Stenosis occurred as a recurrence of some cases treated initially as stenosis, so it is not really a complication of the procedure but an evolution of the pathology.

This was also the first paper that analyzed in an objective way the evolution of patients’ symptoms with sialendoscopy. Even if not being used for all cases yet, ancillary analysis showed an average improvement in visual color scale of symptoms from 9 to 2, and this was statistically significant. We look forward to continuing this analysis to evaluate later outcomes in further studies.

## Conclusions

Sialendoscopy is a challenging procedure with a steep learning progression. We found statistically better outcomes, with higher papilla catheterization rates and more complete examination after the first 50 procedures.

The complication rate was not affected by the learning progression, with statistically the same results between early and late groups of sialendoscopy experience.

## Conflicts of interest

The authors declare no conflicts of interest.
